# Ginsenoside Compound K Attenuates Ox-LDL-Mediated Macrophage Inflammation and Foam Cell Formation *via* Autophagy Induction and Modulating NF-κB, p38, and JNK MAPK Signaling

**DOI:** 10.3389/fphar.2020.567238

**Published:** 2020-09-15

**Authors:** Shan Lu, Yun Luo, GuiBo Sun, XiaoBo Sun

**Affiliations:** ^1^Institute of Medicinal Plant Development, Peking Union Medical College and Chinese Academy of Medical Sciences, Beijing, China; ^2^Institute of Medicinal Plant Development, Beijing Key Laboratory of Innovative Drug Discovery of Traditional Chinese Medicine (Natural Medicine) and Translational Medicine, Beijing, China; ^3^Key Laboratory of Bioactive Substances and Resource Utilization of Chinese Herbal Medicine, Ministry of Education, Beijing, China; ^4^Key Laboratory of Efficacy Evaluation of Chinese Medicine Against Glyeolipid Metabolism Disorder Disease, State Administration of Traditional Chinese Medicine, Beijing, China; ^5^Key Laboratory of New Drug Discovery Based on Classic Chinese Medicine Prescription, Chinese Academy of Medical Sciences, Beijing, China

**Keywords:** atherosclerosis, Ginsenoside compound K, inflammation, autophagy, macrophage

## Abstract

Atherosclerosis is a major reason for the high morbidity and mortality of cardiovascular diseases. Macrophage inflammation and foam cell formation are the key pathological processes of atherosclerosis. Ginsenoside compound K (CK) is a metabolite derived from ginseng. CK has anti atherosclerotic effect, but the molecular mechanism remains to be elucidated. We aim to explore the protective effect of CK against ox-LDL-induced inflammatory responses and foam cells formation *in vitro* and explore its potential mechanisms. Through the results of oil red O staining, Western blot, and qPCR, we found that CK significantly inhibited the foam cell formation, reduced the expression of SR-A1 and increased ABCA1 and ABCG1 expression. In addition, CK increased the number of autophagosomes and upregulated the LC3II/LC3I ratio and the expressions of ATG5 and Beclin-1 but decreased p62 expression. Moreover, CK significantly inhibited the NF-κB, p38, and JNK MAPK signaling pathway. Altogether, CK attenuated macrophage inflammation and foam cell formation *via* autophagy induction and by modulating NF-κB, p38, and JNK MAPK signaling. Thus, CK has potential as a therapeutic drug for atherosclerosis.

## Introduction

Atherosclerosis is the leading cause of coronary heart disease and poses threat to the health of humans worldwide (Virani et al., 2020). It is a complex disease characterized by lipid accumulation within the arterial wall, and vascular cells are involved. Since “inflammation-reaction” theory has been widely accepted in the pathogenesis of atherosclerosis (Ross, 1993), researchers have focused on macrophages. In the early stages of atherosclerosis, macrophages take up a large amount of oxidized low-density lipoprotein (ox-LDL) to produce foam cells. The formation of macrophage foam cells further aggravates atherosclerotic lesions. Theox-LDL stimulates macrophages to produce multiple inflammatory factors, further affecting macrophage cholesterol efflux and aggravating plaque formation (Libby et al., 2002). Although statin (Kühnast et al., 2015) and evolocumab (Giugliano et al., 2017) have been used for atherosclerotic patients in clinic, they are unable to satisfy the needs of these patients. Therefore, foam cell formation and macrophage inflammation are potential strategies in atherosclerosis treatment.

Autophagy is a highly conserved process of degradation and recycling and has been widely reported to play an indispensable role in a variety of biological functions (Nussenzweig et al., 2015). Impaired autophagy, which promoted plaque formation, was found in macrophages in atherosclerosis (Razani et al., 2012). NF-κB and the mitogen-activated protein kinase (MAPK) are important signaling pathways affecting foam cell aggregation and inflammatory response. Inhibition of NF-κB and MAPK signaling attenuates atherosclerosis (Sui et al., 2014). Even more noteworthy is that NF-κB and MAPK have been reported to play a critical role in regulating autophagy. p38 activation promotes cholesterol ester accumulation by suppressing autophagy (Mei et al., 2012) and JNK-mediated macrophage autophagy has been verified in the latest study (Ke et al., 2020). Thus, we speculate that macrophage autophagy could be a promising target for atherosclerosis treatment.

**Figure 1:**
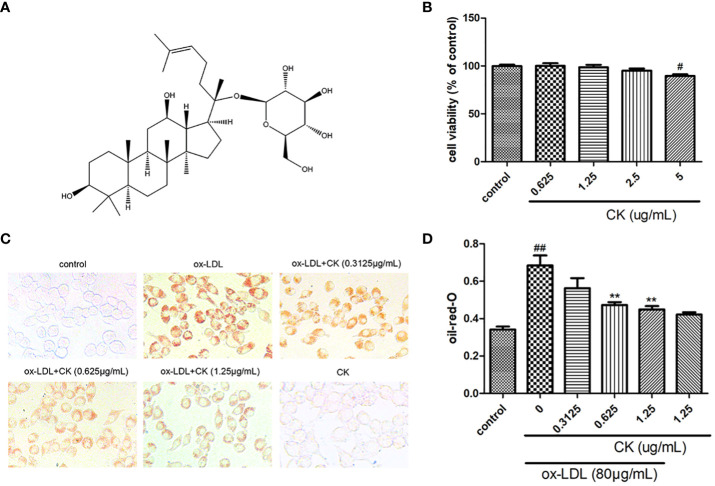
CK inhibited ox-LDL-induced RAW264.7 cells lipid accumulation. RAW264.7 cells were treated with CK at various concentrations for 12 h with or without 80 μg/mL ox-LDL for additional 24 h. **(A)** The chemical formula for CK. **(B)** Cell viability was assayed by the MTT assay. **(C)** Representative images of Oil Red O staining. **(D)** OD value results of oil red O. All data are shown as mean ± SD from three independent experiments with each performed in triplicate. ^#^*P* < 0.05, ^##^*P* < 0.01 vs. control group; **P < 0.01 vs. ox-LDL-treated group. CK, compound K; ox-LDL, oxidized low-density lipoprotein; MTT, (4, 5-dimethylthiazol-2yl-)-2,5-diphenyl tetrazolium bromide.

Ginsenoside compound K (CK, **Figure 1A**) is a ginseng metabolite that can also be biosynthesized (Nan et al., 2020). CK has intriguing pharmacological properties (Yang et al., 2015), such as anti-cancer (Zhang et al., 2018), anti-inflammation (Wang et al., 2019), autophagy induction (Chen et al., 2016), and anti-atherosclerotic effects (Zhou et al., 2016). Moreover, our group previously reported that CK shows protective effects on endothelial cells (Lu et al., 2019). Although CK reportedly inhibits the formation of foam cell in macrophage, its mechanism remains ambiguous and needs to be elucidated.

This present study was aimed to explore the protective effect of CK on ox-LDL-induced inflammatory response and foam cell formation *in vitro* and tried to explore its potential mechanisms for autophagy induction. CK remarkably restrained macrophage foam cell formation and lipid accumulation, as well as the pro-inflammatory cytokines production in RAW 264.7 cells treated with ox-LDL. The mechanism was partly through autophagy induction and the inhibition of NF-κB, P38, and JNK signaling pathways.

## Material and Methods

### Reagents

CK (purity ≥ 98%) was purchased from Shanghai Winherb Medical Science Co., Ltd. (Shanghai, China). Ox-LDL (by copper ion-induced LDL oxidation, MDA = 20 nM) was obtained from Peking Union-Biology Co., Ltd. (Beijing, China). The primary antibodies against P62 and LC3 were purchased from Sigma-Aldrich (St. Louis, MO, USA). The primary antibody against Atg5, Beclin-1, p-p65, IκB, p-IκB, p-IKKβ, IKKβ, p-p38, p38, p-JNK, and JNK was obtained from Cell Signaling Technology (Boston, USA). The β-actin primary antibody was from Abcam (Cambridge, UK). The NF-κB p65 purchased from Santa Cruz Biotechnology (California, USA). The CytoID Autophagy Detection Kit was obtained from Enzo Life Sciences (Farmingdale, NY, USA). Anisomycin was acquired from Selleckchem (Houston, TX, USA). Pyrrolidinedithiocarbamate ammonium (PDTC) was purchased from TargetMol (Shanghai, China). Dimethylsulfoxide (DMSO), oil red O, 3-methyladenine (3-MA) and other conventional reagents were obtained from Sigma-Aldrich (St. Louis, MO, USA).

### Cell Culture and Treatment

RAW264.7 macrophage was obtained from the National Infrastructure of Cell Line Resource (Beijing, China), and cultured in DMEM containing 10% FBS, 100 U/ml penicillin, and 100 μg/ml streptomycin and was maintained at 37°C in 5% CO_2_. RAW264.7 macrophage from passages 5 to 10 were used for the experiments. CK was dissolved in DMSO to form a stock solution and diluted with basic culture medium. The cells were seeded into various plates and pretreated with different concentrations of CK for 12 h with or without the MAPK activator, anisomycin (0.1 μM) or the autophagy inhibitor 3-MA (5 mM). Then cells were stimulated with 80 μg/ml ox-LDL for 24 h. The dosage for CK and ox-LDL was chosen based on previous pharmacodynamics studies (Lu et al., 2019; Luo et al., 2020).

### Oil Red O Staining

Macrophages Oil red O staining was conducted based the method usd in our latest study (Luo et al., 2020). RAW264.7 cells were cultured in 24-well sterile culture plates at 5×10^4^ cells/well. After treatment, the cells were washed with PBS thrice and fixed with 4% paraformaldehyde for 30 min. Then, the cells were subsequently stained with Oil Red O for 1 h and photographed using a light microscope (Olympus, Tokyo, Japan). Then, the absorbance value was detected at 358 nm by a Synergy H1 microplate reader (BioTek, Vermont, USA).

### Transmission Electron Microscopy (TEM)

Macrophage autophagosome detection was performed by using TEM, in accordance with previous report (Luo et al., 2020). Briefly, RAW264.7 cells were seeded into 6-well sterile culture plates at 1×10^6^ cells/well. After all treatment, the gathered cells were fixed in 2.5% glutaraldehyde (TAAB, Berkshire, England) for a whole night. The cells were postfixed in 1% OsO_4_ to increase the membrane contrast and then embedded in epoxypropane through a standard procedure. Ultrathin sections were stained with uranyl acetate and lead citrate and photographed using a JEOL JEM1230 (JEOL Ltd., Tokyo, Japan).

### Autophagosome Formation

Autophagosome formation in macrophages were investigated using a CytoID Autophagy Detection Kit (Enzo Life Sciences, NY, USA) according to our previous study (Luo et al., 2017). Briefly, the cells were inoculated in a 6-well plate at 1×10^6^ cells/well and cultured for 24 h in an incubator at 37 ℃, 5% CO_2_. Different concentrations of CK was then preincubated for 12 h, followed by 80 μg/ml ox-LDL for 24 h. At the end of the treatment, cells were trypsinized. Centrifugation was performed at 1,000 rpm for 5 min to pellet the cells. Cells were washed twice in PBS. A cell sample was resuspended in 250 μl of 1X Assay Buffer. Diluted CYTO-ID^®^ Green stain solution at 250 μl was added to each sample and mixed well. The mixture was incubated for 30 min at room temperature. After treatment, cells were collected by centrifugation and washed with 1X Assay Buffer. Cell pellets were resuspended in 500 μl of fresh 1X Assay Buffer. Samples were analyzed by flow cytometry (BD Biosciences, NJ, USA).

### Flow-Cytometric Analysis

The flow-cytometric analysis was designed to determine the expression of the surface receptor CD36 in macrophages. Briefly, the cells were inoculated in a 6-well plate at 1×10^6^ cells/well and cultured for 24 h in an incubator at 37°C, 5% CO_2_. The cells were preincubated with CK at different concentrations for 12 h and then with 80 μg/ml ox-LDL for 24 h. At the end of the treatment, cells were trypsinized. Centrifugation was performed at 1,000 rpm for 5 min to pellet the cells. The cells were washed with PBS and then incubated with monoclonal antibody against CD36 (BD Biosciences, NJ, USA). They wereincubated for 30 min at 37°C in the dark. After treatment, cells were collected by centrifugation and washed with PBS. Cell pellets were resuspended in 500 μl PBS. Samples were analyzed by flow cytometry (BD Biosciences, NJ, USA).

### RNA Extraction and Quantitative Real-Time PCR (q RT-PCR) Analysis

Total RNA was isolated from cell lysates using Trizol reagent (Invitrogen, USA) following the manufacturer’s instructions. RNA was quantified spectrophotometrically using the NanoDrop system (Thermo Scientific, USA). The isolated RNA was reverse transcribed to cDNA by using PrimeScriptTM RT reagent Kit with gDNA Eraser (TaKaRa, Dalian, China). Quantitative PCR was performed using SYBR Premix Ex TaqTM (TaKaRa, Dalian, China) with a StepOne Plus real-time PCR System (Applied Biosystems, CA, USA). The primer pairs used in this study are listed in **Table 1**. The relative expression level of target genes normalized to GAPDH were calculated using the 2^−ΔΔCT^ method.

**Table 1:** Primers used for quantitative real-time PCR.

Gene	Primer sequence (5′ to 3′)
IL-1β	F: TGCCACCTTTTGACAGTGATGA
	R: TGTGCTGCTGCGAGATTTGA
iNOS	F: CTGCAGCACTTGGATCAGGAACCTG
	R: GGAGTAGCCTGTGTGCACCTGGAA
TNFα	F: AAACCACCAAGTGGAGGAGC
	R: ACAAGGTACAACCCATCGGC
Arg1	F: TGCATATCTGCCAAAGACATCG
	R: TCCATCACCTTGCCAATCCC
Mgl-1	F: ACTTTAGACAACACCACCTCCAA
	R: ATCCTCCACATCCACTTTCAGA
GAPDH	F: CTGCGGCATCCACGAAACT
	R: AGGGCCGTGATCTCCTTCTG
ABCA1	F: GCATTGTCAAGGAGGGGAGAT
	R: CTTCAGGTCAGGGTTGGAGC
ABCG1	F: GTCTGAACTGCCCTACCTACCA
	R: AAAGAAACGGGTTCACATCG

### Western Blot Analysis

Western blots were performed according to reported protocols (Lu et al., 2019). Briefly, total proteins (40 μg) were loaded per lane, separated using 10% SDS-PAGE, and transferred to a nitrocellulose membrane. The membrane was blocked in 5% skim milk at room temperature for at least 2 h and then incubated overnight with the following primary antibodies: P62 and LC3 (1:2,000, Sigma, USA); Atg5, Beclin-1, p-p65, IκB, p-IκB, p-IKKβ, IKKβ, p-p38, p38, p-JNK, and JNK (1:1,000, Cell Signaling Technology, USA); NF-κB p65 (1:200, Santa Cruz, USA); and β-actin (1:2,000, Abcam, USA). After washing thrice with TBST, the membranes were incubated with the corresponding secondary antibody at room temperature for 2 h. Finally, the bands were visualized using an ECL kit (CW0049, CWBIO, Beijing, China).

### Data Analysis

Data were presented as mean ± SD. Statistical analyses were performed using GraphPad Prism 5.0. one-way ANOVA followed by Tukey’s post-hoc test was used for multiple comparison. The statistical significance was set at P <0.05.

## Results

### CK Ameliorated Ox-LDL-Induced Macrophage Derived Foam Cell

**Figure 2:**
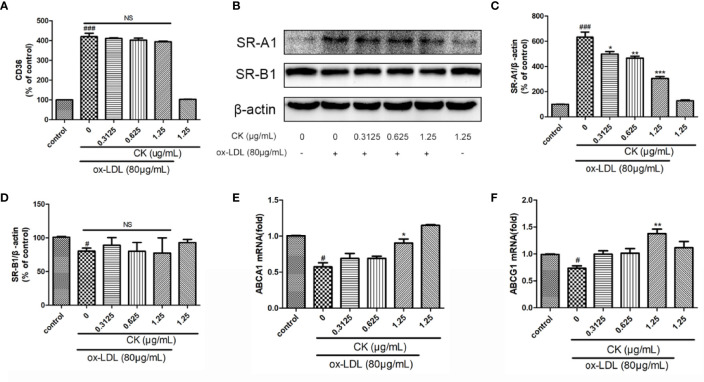
CK inhibited ox-LDL-induced foam cell formation. RAW264.7 cells were treated with CK at various concentrations for 12 h with or without 80 μg/ml ox-LDL for additional 24 h. **(A)** CD36 expression level was tested by flow cytometry. **(B)** The protein expression levels of SR-A1, SR-B1 and β-actin were examined by western blot assay. **(C)** Statistical results of SR-A1 protein level relative to β-actin. **(D)** Statistical results of SR-B1 protein level relative to β-actin. **(E)** The ABCA1 mRNA expression level was determined by RT-PCR assay. **(F)** The ABCG1 mRNA expression level was determined by RT-PCR assay. All data are shown as mean ± SD from three independent experiments with each performed in triplicate. *^#^P* < 0.05, *^###^P* < 0.001 vs. control group; *P < 0.05, **P < 0.01, ***P < 0.001 vs. ox-LDL-treated group. NS, no significance; CK, compound K; ox-LDL, oxidized low-density lipoprotein; SR-A1, scavenger receptor-A1; SR-B1, scavenger receptor-B1; ABCA1, ATP binding cassette subfamily A member 1; ABCG1, ATP binding cassette subfamily G member 1.

Ox-LDL-induced macrophage-derived foam cell is an important factor in the formation of early atherosclerotic plaques (Glass and Witztum, 2001). As shown in **Figure 1B**, treatment with CK less than 2.5 μg/ml did not influence cell viability. Based on the previous study (Luo et al., 2020), we used 80 µg/ml ox-LDL to induce the formation of foam cells in this study. According to oil red O results (**Figures 1C, D**), lipid accumulation was increased by ox-LDL, whereas it was decreased in the group treated with CK at different concentrations. Next, we assessed the effect of CK on lipid internalization. As shown in **Figure 2A**, flow cytometry analysis revealed that CK had no effect on the expression of CD36. However, CK treatment significantly decreased the protein level of SR-A1 (**Figures 2B, C**). To study the effect of CK on cholesterol transportation and metabolism in foam cell (Chistiakov et al., 2017), we evaluated the SR-B1, ABCA1, and ABCG1 expressions. The results demonstrated that 1.25 μg/ml of CK could increase ABCA1 and ABCG1 expressions but had no statistically significant effect on SR-B1 expression (**Figures 2D–F**). Put together, these data indicated that CK can effectively reduce ox-LDL induced foam cell formation.

### CK Reduced Inflammatory Factor Expression in Macrophage

**Figure 3:**
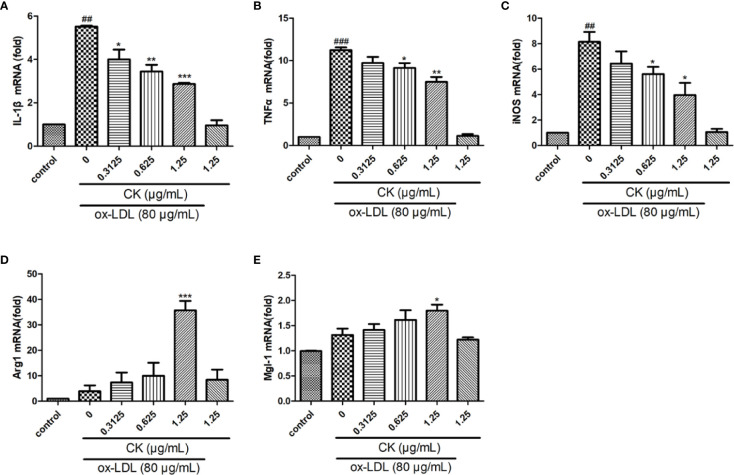
CK regulated ox-LDL-induced macrophages inflammatory factors expression. RAW264.7 cells were treated with CK at various concentrations for 12 h with or without 80 μg/ml ox-LDL for additional 24 h. **(A–E)** IL-1β, TNF-α, iNOS, Arg1, and Mgl-1 mRNA expression levels were tested by RT-PCR assay. All data are shown as mean ± SD from three independent experiments with each performed in triplicate. *^##^P* < 0.01, *^###^P* < 0.001 vs. control group; *P < 0.05, **P < 0.01, ***P < 0.001 vs. ox-LDL-treated group. CK, compound K; ox-LDL, oxidized low-density lipoprotein; IL-1β, interleukin-1β; TNF-α, tumor necrosis factor-α; iNOS, inducible nitric oxide synthase; Arg1, arginase 1; Mgl-1, macrophage galactose-type C-type lectin-1.

Ox-LDL-induced M1 and M2 macrophages phenotype switch is an important event for foam cell formation (van Tits et al., 2011). Thus, we monitored the M1 and M2 macrophage marker expression levels. As shown in **Figures 3A–E**, CK treatment remarkably upregulated Arg1 and Mgl-1 mRNA expression levels but sharply downregulated IL-1β, iNOS, and TNF-α expression levels. Therefore, the results suggested that CK can effectively promote the M2 phenotype switch of macrophages.

### CK Promoted Macrophage Autophagy

**Figure 4:**
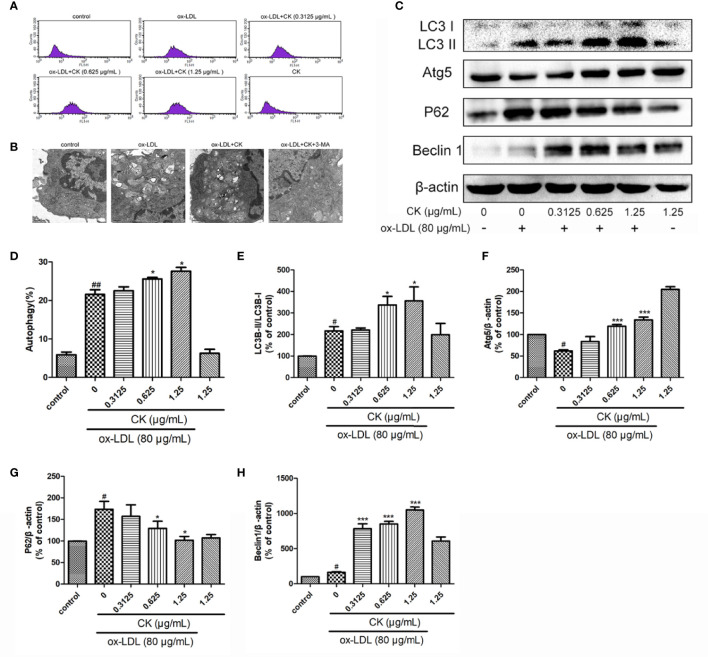
CK promoted ox-LDL induced macrophage autophagy. RAW264.7 cells were treated with CK at various concentrations for 12 h with or without 80 μg/ml ox-LDL for additional 24 h. **(A)** Macrophage autophagosomes were measured by flow cytometry. **(B)** Representative photographs of autophagosomes detected by electron microscope. **(C)** Representative photographs of LC3, Atg5, P62, Beclin-1, and β-actin expressions in ox-LDL-treated macrophages, as examined by western blot assay. **(D)** The percentage of CytoID fluorescence positive cells. **(E–H)** Statistical results of LC3II/LC3I, Atg5, P62 and Beclin-1 expression levels compared with those in the control group. All data are shown as mean ± SD from three independent experiments with each performed in triplicate. *^#^P* < 0.05, *^##^P* < 0.01 vs. control group; *P < 0.05, ***P < 0.001 vs. ox-LDL-treated group. CK, compound K; ox-LDL, oxidized low-density lipoprotein; 3-MA, 3-Methyladenine; Atg5, autophagy related 5; P62, Sequestosome 1.

Results of a previous study demonstrated that macrophage autophagy may be exploited as a promising strategy for atherosclerosis treatment(Shao et al., 2016). Macrophage autophagy plays an important role in inflammation and foam cell formation (Shao et al., 2016; Wu and Lu, 2020), thus, we sought to measure autophagy level in ox-LDL-treated RAW264.7 cells. Autophagy occurred through the formation of autophagosomes. The results showed that CK increased the number of macrophage autophagic vacuoles (**Figures 4A, D**). Moreover, electron microscope result demonstrated that CK increased the number of autophagosomes in macrophage, whereas the autophagy inhibitor, 3-MA reversed the increase of autophagosomes caused by CK (**Figure 4B**). Autophagy activation requires the interaction of several autophagy-related proteins, such as LC3, P62, Atg5, and Beclin-1(Hassanpour et al., 2019). To explore the potential role of CK in these autophagy-related proteins, western blotting assay was performed. As shown in **Figures 4C, E–H**, CK dramatically increased the expressions of Atg5, and Beclin1 and the LC3II/LC3I ratio but notably decreased P62 expression level. Overall, these data suggested that CK promoted macrophage autophagy.

### CK Inhibited NF-κB, P38, and JNK MAPK Pathways

**Figure 5:**
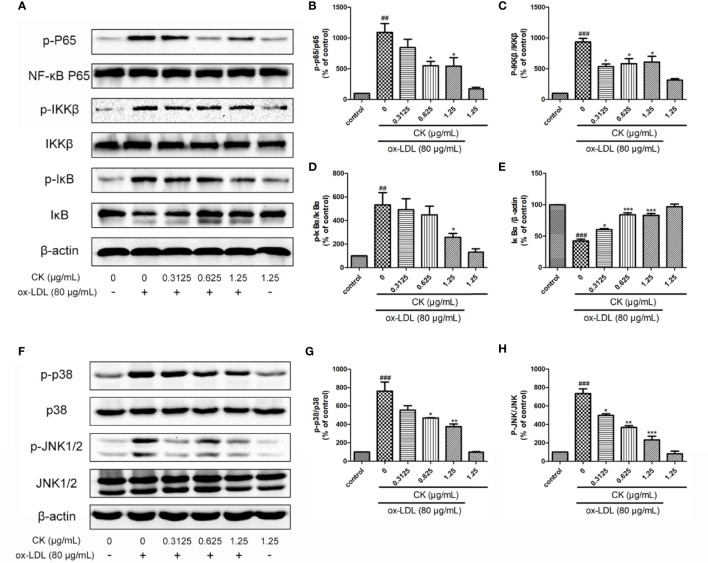
CK inhibited NF-κB, P38, and JNK MAPK pathways. RAW264.7 cells were treated with CK at various concentrations for 12 h with or without 80 μg/ml ox-LDL for additional 24 h. **(A)** The protein expression levels of p-P65, P65, p-IKKβ, IKKβ, p-IκBα, IκBα, and β-actin were examined by western blot assay.**(B–E)** Statistical results of p-P65/P65, p-IKKβ/IKKβ, p-IκBα/β-actin, and IκBα/β-actin expression levels. **(F)** The protein expression levels of p-P38, P38, p-JNK, and JNK were examined by western blot assay. **(G, H)** Statistical results of p-P38/P38 and p-JNK/JNK expression levels. All data are shown as mean ± SD from three independent experiments with each performed in triplicate. *^##^P* < 0.01, *^###^P* < 0.001 vs. control group; *P < 0.05, **P < 0.01, ***P < 0.001 vs. ox-LDL-treated group. CK, compound K; ox-LDL, oxidized low-density lipoprotein; JNK, c-Jun N-terminal kinase; NF-κB, nuclear factor-κB; IKKβ, inhibitor κB kinase β; IκBα, inhibitor of nuclear factor-κBα.

NF-κB, P38, and JNK typical proinflammatory signaling pathways are involved in the expressions of pro-inflammatory genes and autophagy modulation (Yu et al., 2018; Peng et al., 2019), Yet, whether CK modulates these pathways in ox-LDL-induced macrophages remains unknown. We first determined whether CK had an inhibitory effect on NF-κB pathway. As shown in **Figures 5A, B** CK significantly inhibited the phosphorylation of NF-κB P65. NF-κB and IκB bind in the cytoplasm in a stable state. Once stimulated by ox-LDL, IKKβ is activated and phosphorylated, IkB is subsequently phosphorylated and degraded. Then NF-κB is phosphorylated and translocated into the nucleus. Therefore, we investigated the inhibitory effect of CK on IKKβ phosphorylation and IκBα degradation and phosphorylation in RAW264.7 macrophages stimulated with ox-LDL. Our data showed that CK significantly prevented the phosphorylation of IKKβ and suppressed IκBα degradation and phosphorylation (**Figures 5A, C–E**). In addition, we next assessed the effect of CK on P38 and JNK pathways. As shown in **Figures 5F–H**, CK noticeably suppressed the expression of the phosphorylated forms of P38 and JNK. Altogether, these data suggested that CK had obvious inhibitory effects on NF-κB, P38, and JNK pathways.

### CK Mediated-Autophagy and Anti-Inflammation Were Abolished by NF-κB, P38, and JNK MAPK Activation

**Figure 6:**
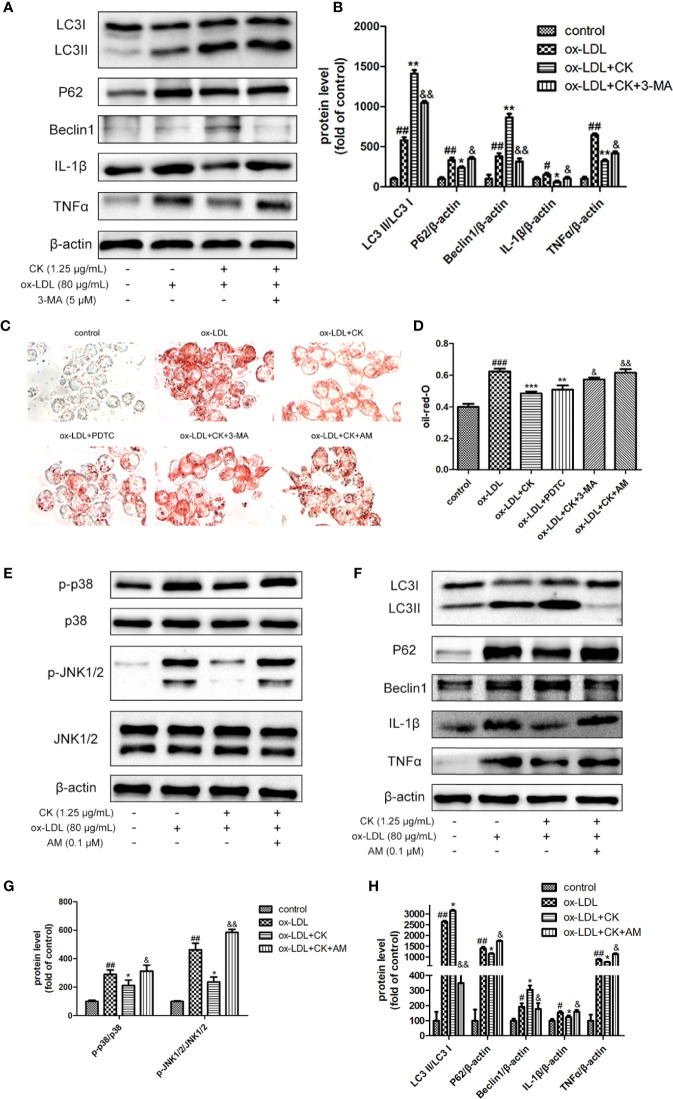
CK mediated-autophagy and anti-inflammation were abolished by NF-κB, P38, and JNK MAPK activation. RAW264.7 cells were treated with CK (1.25 μg/mL) for 12 h with or without the NF-κB inhibitor, PDTC (10 μM) or the MAPK activator, anisomycin (0.1 μM) or the autophagy inhibitor 3-MA (5 mM). Then cells were stimulated with 80 μg/mL ox-LDL for 24 h. **(A)** The protein expression levels of LC3, Beclin-1, P62, IL-1β, TNF-α, and β-actin were examined by western blot assay. **(B)** Statistical results of LC3II/LC3I, Beclin-1, P62, IL-1β, and TNF-α expression levels. **(C)** Representative images of Oil Red O staining. **(D)** OD value results of oil red O. **(E)** Representative Western blot analysis of phosphorylated and total p38, and JNK was performed. **(F)** The expression levels of LC3, Beclin-1, P62, IL-1β, TNF-α, and β-actin were detected by Western blot analysis. **(G)** Densitometric analysis was used to quantify the levels of p-p38, p-JNK. **(H)** Statistical results of LC3II/LC3I, Beclin-1, P62, IL-1β, and TNF-α expression levels. All data are shown as mean ± SD from three independent experiments with each performed in triplicate. *^#^P* < 0.05, *^##^P* < 0.01, *^###^P* < 0.001 vs. control group; *P < 0.05, **P < 0.01, ***P < 0.001 vs. ox-LDL-treated group; *^&^P* < 0.05, *^&&^P* < 0.01 vs. ox-LDL and CK treatment group. CK, compound K; PDTC, pyrrolidinedithiocarbamate ammonium; 3-MA, 3-Methyladenine; AM, anisomycin.

We used 3-MA as an autophagy inhibitor to demonstrate the involvement of autophagy in the inhibitory effects of CK on foam cell formation and inflammatory response. As shown in **Figures 6A, B**, compared with the CK group, the autophagy was successfully abated by 3-MA treatment, as confirmed by the downregulation of Beclin-1 expression and LC3II/LC3I ratio and the upregulation of P62 expression. In addition, after 3-MA pretreatment, the anti-inflammatory effect of CK was diminished, as manifested by the increased expressions of IL-1β and TNFα. Moreover, foam cell formation significantly increased after 3-MA treatment, as lipid droplets were clearly viewed in **Figures 6C, D**. These findings suggest that CK attenuated the formation of foam cells and inflammatory response *via* autophagy.

NF-κB and MAPK play a critical role in regulating autophagy. The autophagy level in the RAW264.7 cells decreased significantly following the inhibition of the NF-κB pathway (Liang et al., 2017). Moreover, p38 activation promoted cholesterol ester accumulation by suppressing autophagy (Mei et al., 2012) and JNK-mediated macrophage autophagy has been verified in the latest study (Ke et al., 2020). However, whether CK induces autophagy and reduces foam cell formation and inflammation by inhibiting NF-κB and MAPK signaling pathways remains unknown. To confirm the involvement of these pathways in the effects of CK on autophagy, foam cell formation, and inflammatory response, PDTC (NF-κB inhibitor) or anisomycin (P38 and JNK MAPK activator) was applied before CK treatment. As shown in **Figures 6C, D**, PDTC reduced macrophage lipid droplets and inhibited the formation of foam cells. In contrast, anisomycin partially abolished the inhibition effects of CK on foam cell formation. In addition, anisomycin reversed the inhibition effect of CK on P38 and JNK MAPK pathway (**Figures 6E, F**). Moreover, the addition of anisomycin before CK treatment weakened the induction effect of CK on autophagy, as manifested by the decreased expressions of LC3 II/LC3 I and Beclin-1 and the increased expression of P62 (**Figures 6G, H**). After anisomycin pretreatment, the anti-inflammatory effect of CK was diminished, as manifested by the increased expressions of IL-1β and TNFα. To sum up, CK induced autophagy and reduced foam cell formation and inflammation by inhibiting the NF-κB and MAPK signaling pathways.

## Discussion

Although statins and other new atherosclerosis therapeutic agents (Kühnast et al., 2015) have been successfully applied, new drugs are needed to meet the unmet clinical demand and to address the high morbidity and mortality due to atherosclerosis (Virani et al., 2020). Saponins, a gift from nature, have been widely studied in recent decades for its high biological activity (Marciani, 2018). Ginsenoside CK, isolated from ginseng, known for its availability, safety, and procurability (Wang et al., 2019; Li et al., 2020; Nan et al., 2020). Although CK reportedly showed anti-atherosclerosis effect (Zhou et al., 2016) in ApoE^-/-^ mice, its mechanism remains ambiguous and needs to be elucidated. The present study demonstrated that CK remarkably restrained macrophage foam cell formation and lipid accumulation in RAW 264.7 cells treated with ox-LDL. CK also inhibited the production of pro-inflammatory cytokines. The mechanism is partly autophagy induction and modulation of NF-κB, P38, and JNK signaling.

As a major component of atherosclerotic lesions, foam cells play a vital role in the development of atherosclerosis (Chistiakov et al., 2017). In this study, we first investigated the inhibitory effects of CK on ox-LDL-induced RAW264.7 foam cell formation. The effects of ox-LDL on lipid accumulation were partially blocked by CK, which was in accordance with the results of a previous study (Zhou et al., 2016). Cholesterol metabolism is involved in foam cell formation (McLaren et al., 2011; Bories and Leitinger, 2017). In general, increased intracellular cholesterol uptake and reduced cholesterol efflux lead to the accumulation of large lipid droplets in macrophage foam cells (Shen D. et al., 2019). Macrophage scavenger receptors (SRs), including SR-A1 and CD36, mediate intracellular cholesterol uptake. Cholesterol efflux is mostly mediated by ATP-binding cassette transporters ABCA1 and ABCG1, as well as SR-B1, for reverse cholesterol transport (Tall and Yvan-Charvet, 2015). CK effectively reduced the protein expression of SR-A1 and increased the mRNA expressions of ABCA1 and ABCG1. Collectively, these findings suggested that CK can effectively reduce foam cell formation by downregulating cholesterol uptake and upregulating cholesterol efflux, further confirming the anti-atherosclerosis effect of CK.

Macrophage inflammation plays an important role in the development of atherosclerosis. When stimulated by ox-LDL, the transformation of macrophages into foam cells leads to an inflammatory response (Cybulsky et al., 2016). In addition, the macrophage phenotype affects the development of atherosclerosis. M1 macrophages play a pro-inflammatory role in aggravating the development of atherosclerosis, whereas M2 macrophages play an anti-inflammatory role in reducing atherosclerotic lesions (De Paoli et al., 2014; Tabas and Bornfeldt, 2016). CK inhibited the expressions of inflammatory mediators, such as IL-1β, iNOS, and TNF-α. However, CK increased the gene expressions of Arg1 and Mgl-1, which are associated with the anti-inflammatory phenotype of M2 macrophages. This finding implied that CK stimulated macrophages into anti-inflammation M2 phenotype to repress inflammation.

Moderate autophagy can inhibit the formation and development of atherosclerotic plaques. Our previous studies have demonstrated that macrophage autophagy can reduce the formation of foam cells and the secretion of inflammatory factors and promote the transformation of macrophages to the M2 phenotype (Luo et al., 2020). In addition, the genetic knockout of the key autophagy gene Atg5 can significantly inhibit cholesterol leakage and promote the formation of foam cells (Singh et al., 2009). During autophagy, cytoplasmic LC3-I is converted to LC3-phosphatidyl ethanolamine (LC3-II). Thus, the LC3-II/LC3-I ratio is often used as a quantitative indicator of autophagy (Wu and Lu, 2020). Beclin-1 and p62 are upregulated and downregulated respectively in autophagy, which have important functions in autophagy regulation and are commonly used markers for autophagy detection (Lamark et al., 2017). Our data demonstrated that CK increased the LC3-II/LC3-I ratio and Beclin-1 and Atg5 expressions but decreased the expression of P62, thereby suggesting that CK reduced the formation of foam cells and promoted M2 macrophage phenotype partly through autophagy induction.

NF-κB and MAPK are also important signaling pathways that affect foam cell aggregation and inflammatory response (Kim and Kim, 2019; Shen W. et al., 2019). Previous studies have demonstrated that NF-κB is a key regulator of macrophage inflammation, and NF-κB inhibition reduces foam cell formation (Plotkin et al., 2017; Islam et al., 2018; Adam et al., 2019; Kim and Kim, 2019). The current study showed that CK significantly inhibited phosphorylation of IKKβ and suppressed IκBα degradation and phosphorylation, thereby reducing the phosphorylation of NF-κB P65. Activation of the p38MAPK pathway inhibited the cholesterol efflux and the expressions of ABCA1, ABCG1, and SR-B1 in ox-LDL-induced macrophages (Cheng et al., 2016). P38 MAPK and JNK inhibitor significantly decreased foam cell formation (Geng et al., 2018). Importantly, He et al. revealed that p38α MAPK plays a direct and essential role in relieving autophagic control in response to an inflammatory signal (He et al., 2018). Consistently, the study showed the substantial decrease of P38 and JNK phosphorylation in ox-LDL-induced macrophages after CK treatment, resulting in the alleviation of inflammation and the reduction of lipid accumulation.

In summary, the results of the present study indicated that CK remarkably restrained macrophage foam cell formation and lipid accumulation, as well as the pro-inflammatory cytokines production in RAW 264.7 cells treated with ox-LDL. However, the limitation of this study is the lack of effective positive controls. The underlying mechanism was partly through autophagy induction and the inhibition of the NF-κB, P38 and JNK signaling pathways. CK is promising for use in the inhibition of inflammation and lipid accumulation in macrophages to inhibit atherosclerosis development.

## Data Availability Statement

The raw data supporting the conclusions of this article will be made available by the authors, without undue reservation, to any qualified researcher.

## Author Contributions

SL, GS, and XS conceived and designed the experiments. SL and YL performed the experiments. YL collected and analyzed the data. SL wrote original draft. GS revised the manuscript. GS and XS supervised manuscripts. XS validated the manuscript, wrote review and edited.

## Funding

This work was supported by Key Laboratory of new drug discovery based on Classic Chinese medicine prescription, Chinese Academy of Medical Sciences (No. 2018PT35030), the Drug Innovation Major Project (No. 2018ZX09711001-009), and the National Natural Science Foundation of China (No. 81891012).

## Conflict of Interest

The authors declare that the research was conducted in the absence of any commercial or financial relationships that could be construed as a potential conflict of interest.

## Correction Note

A correction has been made to this article. Details can be found at: 10.3389/fphar.2025.1499242.
