# Corrigendum: Ginsenoside compound K attenuates ox-LDL-mediated macrophage inflammation and foam cell formation *via* autophagy induction and modulating NF-kB, p38, and JNK MAPK signaling

**DOI:** 10.3389/fphar.2024.1452605

**Published:** 2024-07-23

**Authors:** Shan Lu, Yun Luo, GuiBo Sun, XiaoBo Sun

**Affiliations:** ^1^ Institute of Medicinal Plant Development, Peking Union Medical College and Chinese Academy of Medical Sciences, Beijing, China; ^2^ Institute of Medicinal Plant Development, Beijing Key Laboratory of Innovative Drug Discovery of Traditional Chinese Medicine (Natural Medicine) and Translational Medicine, Beijing, China; ^3^ Key Laboratory of Bioactive Substances and Resource Utilization of Chinese Herbal Medicine, Ministry of Education, Beijing, China; ^4^ Key Laboratory of Efficacy Evaluation of Chinese Medicine Against Glyeolipid Metabolism Disorder Disease, State Administration of Traditional Chinese Medicine, Beijing, China; ^5^ Key Laboratory of New Drug Discovery Based on Classic Chinese Medicine Prescription, Chinese Academy of Medical Sciences, Beijing, China

**Keywords:** atherosclerosis, Ginsenoside compound K, inflammation, autophagy, macrophage

In the published article, there was an error in Figure 1 as published. In Figure 1C, the representative pictures in the control and CK groups were the same as in one of our previously published papers. The corrected Figure 1 and its caption appear below.

**FIGURE 1:**
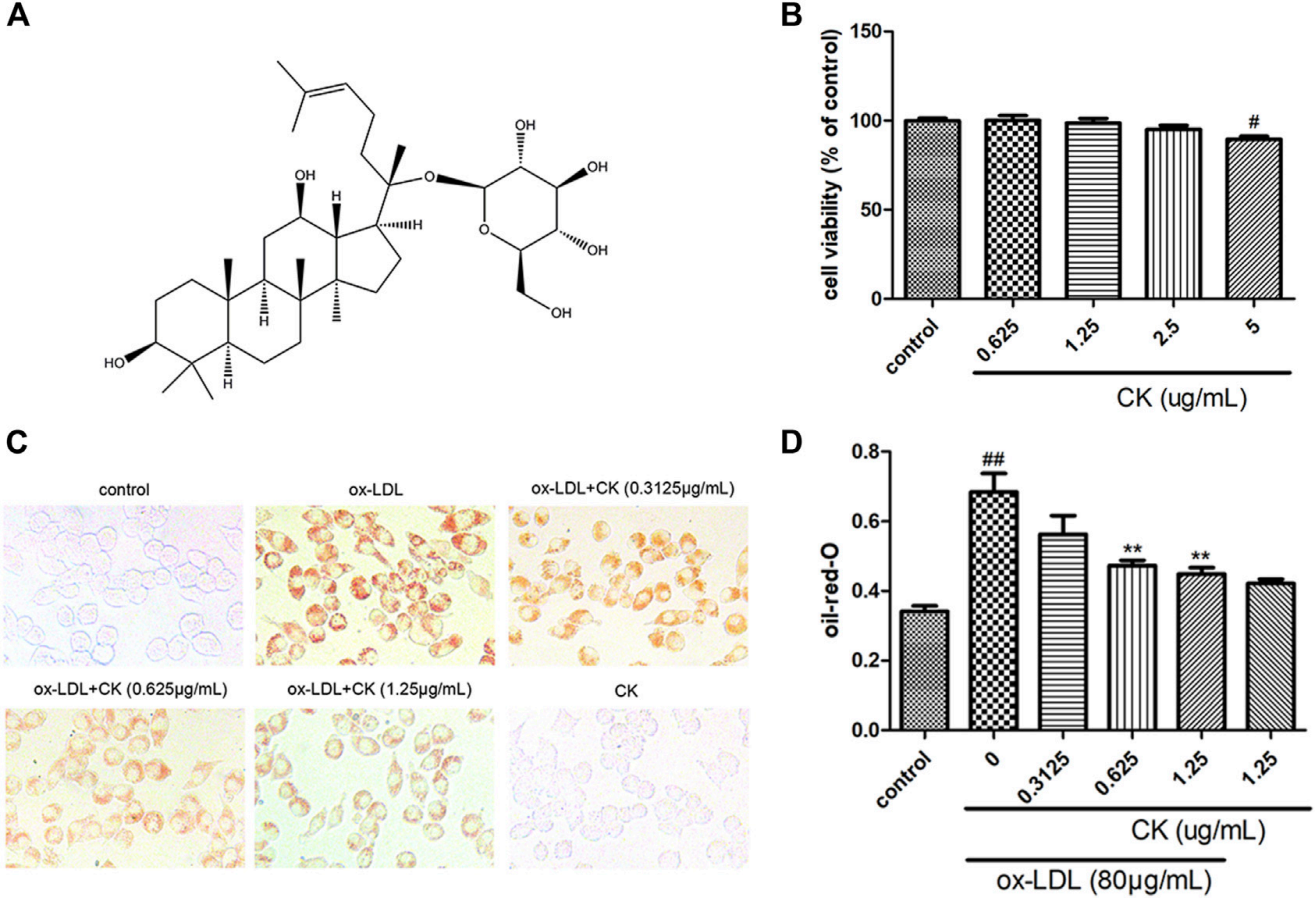
CK inhibited ox-LDL-induced RAW264.7 cells lipid accumulation. RAW264.7 cells were treated with CK at various concentrations for 12 h with or without 80 mg/mL ox-LDL for additional 24 h. **(A)** The chemical formula for CK. **(B)** Cell viability was assayed by the MTT assay. **(C)** Representative images of Oil Red O staining. **(D)** Oil red O positive area was measured by ImageJ software. All data are shown as mean ± SD from three independent experiments with each performed in triplicate. ^#^ *p* < 0.05, ^##^ *p* < 0.01 vs. control group; **p < 0.01 vs. ox-LDL-treated group. CK, compound K; ox-LDL, oxidized low-density lipoprotein; MTT, (4, 5-dimethylthiazol-2yl-)-2,5-diphenyl tetrazolium bromide.

The authors apologize for this error and state that this does not change the scientific conclusions of the article in any way. The original article has been updated.

