# Correction: Ginsenoside compound K attenuates Ox-LDL-mediated macrophage inflammation and foam cell formation via autophagy induction and modulating NF-κB, p38, and JNK MAPK signaling

**DOI:** 10.3389/fphar.2025.1499242

**Published:** 2025-06-17

**Authors:** Shan Lu, Yun Luo, GuiBo Sun, XiaoBo Sun

**Affiliations:** ^1^ Institute of Medicinal Plant Development, Peking Union Medical College and Chinese Academy of Medical Sciences, Beijing, China; ^2^ Institute of Medicinal Plant Development, Beijing Key Laboratory of Innovative Drug Discovery of Traditional Chinese Medicine (Natural Medicine) and Translational Medicine, Beijing, China; ^3^ Key Laboratory of Bioactive Substances and Resource Utilization of Chinese Herbal Medicine, Ministry of Education, Beijing, China; ^4^ Key Laboratory of Efficacy Evaluation of Chinese Medicine Against Glyeolipid Metabolism Disorder Disease, State Administration of Traditional Chinese Medicine, Beijing, China; ^5^ Key Laboratory of New Drug Discovery Based on Classic Chinese Medicine Prescription, Chinese Academy of Medical Sciences, Beijing, China

**Keywords:** atherosclerosis, ginsenoside compound K, inflammation, autophagy, macrophage

In the published article, there was an error in the legend for Figure 1 as published. In Figure 1D, it was an error to state that oil red O positive area was measured by Image J software. The corrected legend appears below.

**FIGURE 1:**
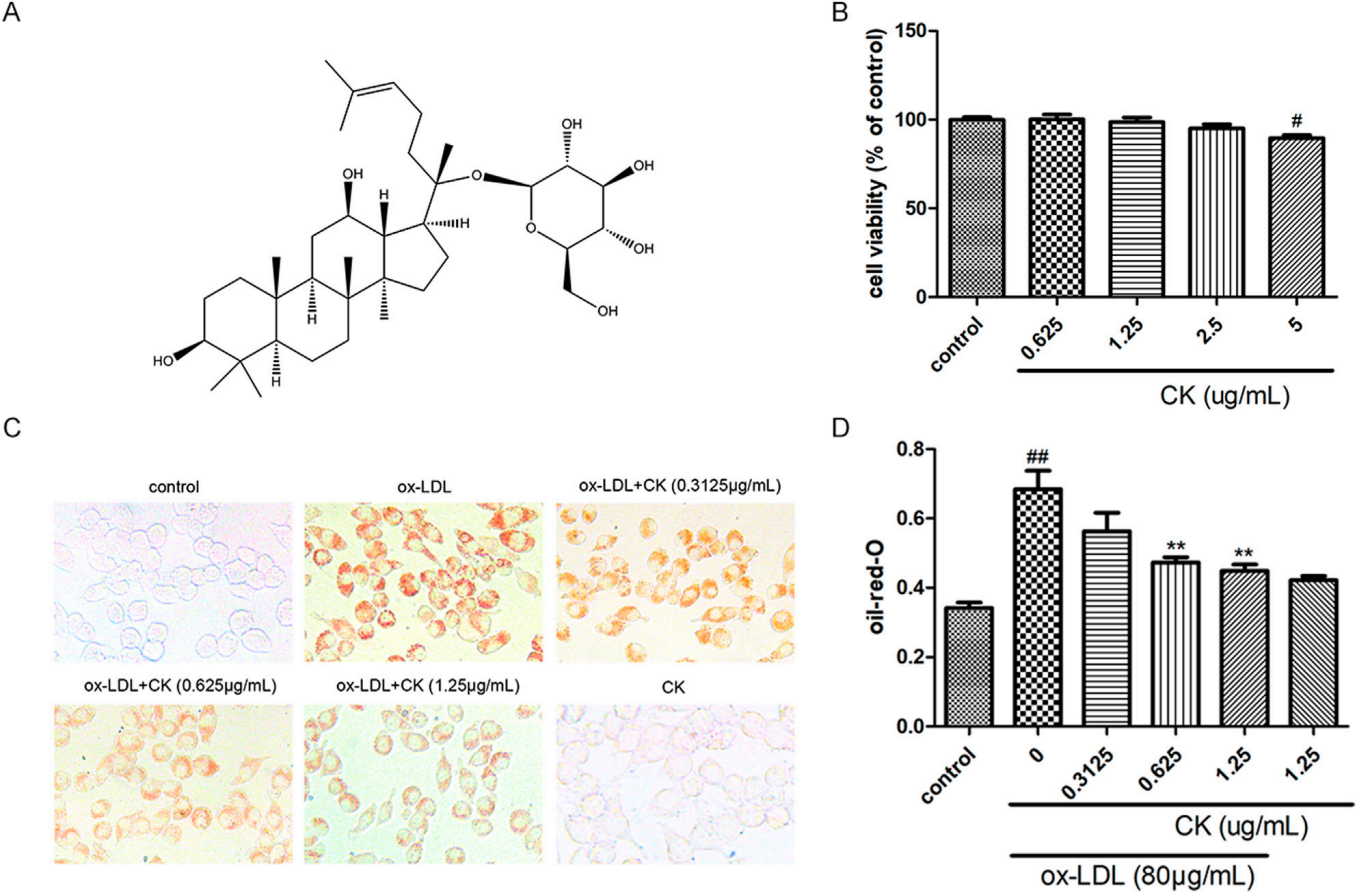
CK inhibited ox-LDL-induced RAW264.7 cells lipid accumulation. RAW264.7 cells were treated with CK at various concentrations for 12 h with or without 80 μg/mL ox-LDL for additional 24 h. **(A)** The chemical formula for CK. **(B)** Cell viability was assayed by the MTT assay. **(C)** Representative images of Oil Red O staining. **(D)** OD value results of oil red O. All data are shown as mean ± SD from three independent experiments with each performed in triplicate. ^#^ *P* < 0.05, ^*##*^ *P* < 0.01 vs. control group; **P < 0.01 vs. ox-LDL-treated group. CK, compound K; ox-LDL, oxidized low-density lipoprotein; MTT, (4, 5-dimethylthiazol-2yl-)-2,5-diphenyl tetrazolium bromide.

“CK inhibited ox-LDL-induced RAW264.7 cells lipid accumulation. RAW264.7 cells were treated with CK at various concentrations for 12 h with or without 80 μg/mL ox-LDL for additional 24 h. **(A)** The chemical formula for CK. **(B)** Cell viability was assayed by the MTT assay. **(C)** Representative images of Oil Red O staining. **(D)** OD value results of oil red O. All data are shown as mean ± SD from three independent experiments with each performed in triplicate. ^#^ *P* < 0.05, ^*##*^ *P* < 0.01 vs. control group; **P < 0.01 vs. ox-LDL-treated group. CK, compound K; ox-LDL, oxidized low-density lipoprotein; MTT, (4, 5-dimethylthiazol-2yl-)-2,5-diphenyl tetrazolium bromide.”

In the published article, there was an error in Figure 1 as published. In Figure 1C, the representative picture of the CK group was updated with the correct one. The corrected Figure 1 and its caption appear above.

In the published article, there was an error in the legend for **Figure 6** as published. In **Figure 6D**, it was an error to state that oil red O positive area was measured by Image J software. The corrected legend appears below.

“CK mediated-autophagy and anti-inflammation were abolished by NF-κB, P38, and JNK MAPK activation. RAW264.7 cells were treated with CK (1.25 μg/mL) for 12 h with or without the NF-κB inhibitor, PDTC (10 μM) or the MAPK activator, anisomycin (0.1 μM) or the autophagy inhibitor 3-MA (5 mM). Then cells were stimulated with 80 μg/mL ox-LDL for 24 h. **(A)** The protein expression levels of LC3, Beclin-1, P62, IL-1β, TNF-α, and β-actin were examined by western blot assay. **(B)** Statistical results of LC3II/LC3I, Beclin-1, P62, IL-1β, and TNF-α expression levels. **(C)** Representative images of Oil Red O staining. **(D)** OD value results of oil red O. **(E)** Representative Western blot analysis of phosphorylated and total p38, and JNK was performed. **(F)** The expression levels of LC3, Beclin-1, P62, IL-1β, TNF-α, and β-actin were detected by Western blot analysis. **(G)** Densitometric analysis was used to quantify the levels of p-p38, p-JNK. **(H)** Statistical results of LC3II/LC3I, Beclin-1, P62, IL-1β, and TNF-α expression levels. All data are shown as mean ± SD from three independent experiments with each performed in triplicate. ^*#*^ *P* < 0.05, ^*##*^ *P* < 0.01, ^*###*^ *P* < 0.001 vs. control group; *P < 0.05, **P < 0.01, ***P < 0.001 vs. ox-LDL-treated group; ^*&*^ *P* < 0.05, ^*&&*^ *P* < 0.01 vs. ox-LDL and CK treatment group. CK, compound K; PDTC, pyrrolidinedithiocarbamate ammonium; 3-MA, 3-Methyladenine; AM, anisomycin.”

The original version of this article has been updated.

